# Stability of soil bacteria in undisturbed soil and continuous maize cultivation in Northern Thailand

**DOI:** 10.3389/fmicb.2023.1285445

**Published:** 2023-11-03

**Authors:** Noppol Arunrat, Chakriya Sansupa, Sukanya Sereenonchai, Ryusuke Hatano

**Affiliations:** ^1^Faculty of Environment and Resource Studies, Mahidol University, Nakhon Pathom, Thailand; ^2^Department of Biology, Faculty of Science, Chiang Mai University, Chiang Mai, Thailand; ^3^Laboratory of Soil Science, Research Faculty of Agriculture, Hokkaido University, Sapporo, Japan

**Keywords:** maize cultivation, rotational shifting cultivation, undisturbed soil, soil bacteria, 16S rRNA gene

## Abstract

Rotational shifting cultivation (RSC) in Northern Thailand serves the dual purpose of ensuring food security and meeting economic goals through maize cultivation. However, the research question remains: Does the dynamics of soil bacterial communities differ between maize monoculture and RSC fields with continuous fallow throughout the season? Therefore, the objective of this study was to investigate and compare the variation of soil bacterial communities in maize monoculture and fallow RSC fields. A continuous 5-year fallow field (undisturbed soil; CF-5Y) and a continuous 5-year maize cultivation field (M-5Y) in Mae Chaem District, Chiang Mai Province, Northern Thailand, were selected due to their similarities in microclimate, topography, and the 5-year duration of different field activities. Over the span of a year, we collected soil samples from the surface layer (0–2 cm depth) at both sites. These collections occurred at 3-month intervals, starting from March 2022 (summer season) and followed by June (rainy season), September (rainy season), December (winter season), and March 2023 (summer season). Soil bacterial diversity and composition were analyzed using 16S rRNA gene-based metagenomic analysis. The results found that undisturbed soil over a 5-year period exhibited more stability in the richness and diversity of bacteria across seasons compared with M-5Y. Notably, fertilizer application and tillage practices in M-5Y can enhance both the diversity and richness of soil bacteria. In terms of bacterial abundance, Proteobacteria prevailed in CF-5Y, while Actinobacteria dominated in M-5Y. At the genus level, Candidatus *Udaeobacter* dominated during the summer and winter seasons in both CF-5Y and M-5Y sites. Interestingly, during the rainy season, the dominant genus shifted to *Bacillus* in both CF-5Y and M-5Y fields. The soil bacterial community in M-5Y was strongly influenced by organic matter (OM) and organic carbon (OC). In contrast, in CF-5Y, there was no correlation between soil properties and the soil bacterial community, likely due to the lower variation in soil properties across seasons. β-Glucosidase was the dominant enzyme in both CF-5Y and M-5Y sites, and it showed a positive correlation with OM and OC. Further studies should continue to investigate soil bacteria dynamics, considering the changes in land management practices.

## 1. Introduction

Soil microorganisms play a crucial role in the dynamic transformation of soil nutrients (Schulz et al., 2013), organic matter transformation, the enhancement of plant productivity, and the control of soil-borne diseases (Pieterse et al., 2016). However, soil microorganisms are highly sensitive to soil disturbance and management practices, which can lead to changes in their functionality (Cordovez et al., 2019). Changes in land use, specifically alterations in vegetation cover, can significantly impact soil physicochemical properties and drive shifts in microbial community composition (Szoboszlay et al., 2017; Meng et al., 2019). The intensification of land use, such as the conversion of natural vegetation to arable agriculture, has been associated with a substantial loss of soil organic carbon (SOC) by up to 44.9% (Arunrat et al., 2022a), primarily due to the loss of vegetation cover, litter, and root biomass (Whitbread et al., 1999; Shahzad et al., 2018). These changes ultimately lead to a decrease in microbial populations and a reduction in soil microbial diversity (Williams et al., 2017; Newbold et al., 2018), resulting in degraded soil ecosystems and long-term decline in soil fertility (Gouda et al., 2018). Therefore, the identification and quantification of soil microorganisms are essential for assessing soil quality status, which can contribute to maintaining soil nutrient levels and improving crop productivity (Schloter et al., 2003).

Shifting cultivation, also known as swidden farming or slash-and-burn farming, is a widely practiced agricultural method in the highlands of Southeast Asia (Rerkasem and Rerkasem, 1995). It is deeply rooted in the cultural heritage of indigenous communities. The practice involves a cyclic rotation of farming areas, encompassing conversion, cultivation, harvesting, burning, and fallow periods lasting for 5–15 years (Kleinman et al., 1995). In Northern Thailand, rotational shifting cultivation (RSC) is a traditional farming practice among the inhabitants of mountainous regions (Arunrat et al., 2022b). RSC involves managing fallow cycles where one area is temporarily cultivated and subsequently left fallow to allow vegetation and soil fertility to recover, while the farmers move on to another area (Arunrat et al., 2023a). Fire is utilized as a land preparation tool during the conversion process, altering soil characteristics depending on the severity and intensity of the burn (Caon et al., 2014). A study conducted by Wapongnungsang et al. (2021) examined soil properties in shifting agricultural areas over 10 and 15 years. The findings indicated that pH, as well as the availability of phosphorus (P) and nitrogen (N), increased in the burned regions compared with the pre-burned areas. Moreover, previous research suggests that shifting agriculture not only affects soil physical structure and biochemical characteristics but also alters soil microbial communities due to the heat generated during combustion (Certini, 2005; Barreiro and Díaz-Raviña, 2021), potentially influencing the survival and growth of bacteria. *Bacillus* sp. have been commonly found in burned swidden areas (Smith et al., 2008; Miah et al., 2010). However, when comparing bacterial communities in shifting agriculture areas with a 13-year-old reforested area, distinct differences were observed. Cultivated areas showed the presence of *Pseudomonas diminuta* and *Shigella*, whereas forested areas contained *Bacillus firmus* and *Xanthomonas*, with variations in bacterial strains linked to soil properties (Miah et al., 2014). During the fallow period, the field accumulates organic matter (OM) inputs from litter and roots (Sarkar et al., 2015), which significantly impacts soil microbial communities (Kim et al., 2020).

Maize (*Zea mays* Lin.) is a vital food and industrial crop, ranking among the most widely cultivated crops worldwide. In 2022, Thailand exported approximately 977.15 tons of maize and imported approximately 1.4 million tons, indicating a high demand for maize in the country. Maize is predominantly used in the animal feed industry, which contributes to its economic significance (Ekasingh et al., 2004). Thai farmers are increasingly shifting toward maize production for animal feed due to its easier management, shorter growth cycle, and lower water requirements compared with rice cultivation. However, continuous maize cultivation in certain areas has led to challenges such as soil degradation (Moebius-Clune et al., 2011) and a decline in biodiversity due to the prolonged cultivation of a single plant variety. Additionally, soil fertility limitations often impact maize development and yield (Essel et al., 2020). The growth of maize is influenced by various factors, including soil microorganisms, organic fertilizer treatments, chemical fertilizers, and soil nutrient availability. Previous studies have compared the response of bacteria in maize-growing areas under organic fertilizer application versus no organic fertilizer application, revealing that the use of organic fertilizer can positively impact maize growth (Wu et al., 2021). Furthermore, *Mycobacterium phlei* was identified as an efficient bacterial strain for enhancing maize growth (Egamberdiyeva, 2007). In Northeast China, Yang et al. (2022) demonstrated that maize cropping and continuous cropping of alfalfa increased soil bacterial alpha diversity compared with meadow cropping. This can be attributed to soil management practices, including tillage operations, which promote increased decomposition and mineralization of available nutrients, thereby influencing the structure of soil bacterial communities.

However, the extensive cultivation of maize in Northern Thailand, driven by high price incentives, has resulted in a reduction in the number of RSC fields. As a consequence, there is a lack of direct evidence regarding the dynamics of soil bacterial communities under maize monoculture compared with RSC fields with continuous fallow. Therefore, the objective of this study was to investigate and compare the variation of soil bacterial communities in areas of maize monoculture and RSC fields with a continuous 5-year fallow period. Our hypothesis posited that 5 years of undisturbed soil would lead to a more stable community composition compared with monoculture.

## 2. Materials and methods

### 2.1. Study areas

**Figure 1:**
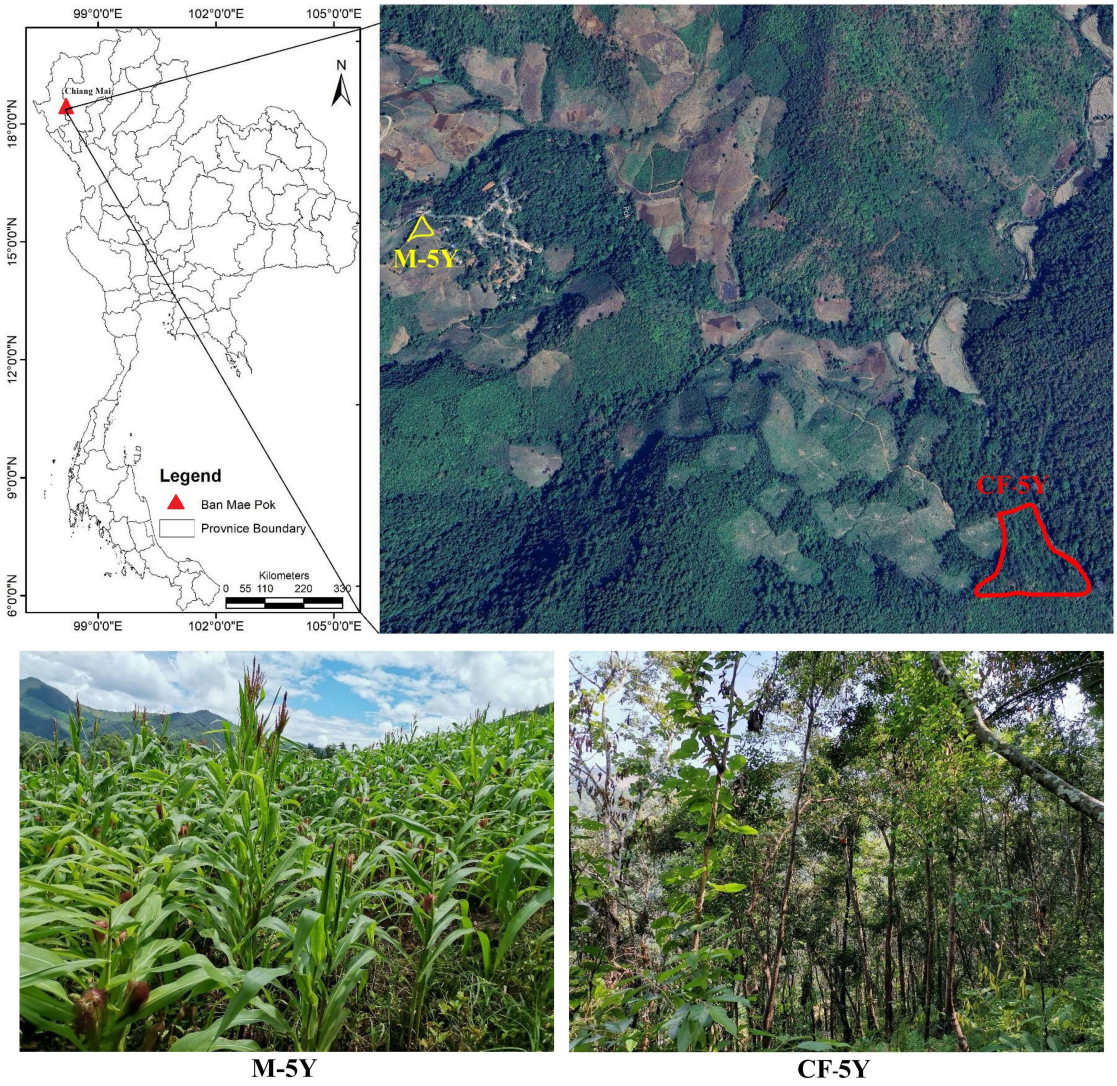
Study areas. The aerial image was captured from Google Earth on 29 August 2023. The photos were taken by Noppol Arunrat.

The study sites were situated in Ban Mae Pok, Ban Thab Subdistrict, Mae Chaem District, Chiang Mai Province, Northern Thailand (Figure 1). Average weather data, including maximum, minimum, and mean temperatures, precipitation, and the number of rainy days, for the study period (March 2022 to March 2023) were obtained from the Thai Meteorological Department, sourced from Doi Ang Khang and Mueang Chiang Mai stations (see Supplementary Table S1; Supplementary Figure S1). A continuous 5-year fallow field (CF-5Y) and a continuous 5-year maize cultivation field (M-5Y) were selected due to their similarity in microclimate, topography, and 5-year duration of different field activities. The CF-5Y field (18°23′3”N, 98°11′49″E; elevation 659 m a.s.l) was used for cultivating upland rice, and after the harvest, it has been left abandoned for 5  years. The M-5Y field (18°23′30”N, 98°10′52″E; elevation 823 m a.s.l) was previously abandoned land from upland rice cultivation, and it has been converted into a maize field for 5 years. Tillage is typically performed in approximately May, which marks the early rainy season. This practice involves plowing to a depth of 20–30 cm. Subsequently, maize seeds were sown in June, and the harvest was carried out in November. Chemical fertilizer was applied only once at the flowering stage during August (40–50 days after sowing). Nitrogen fertilizer was applied at a rate of approximately 25.5 to 30.2 kg N ha^−1^ year^−1^, while phosphorus fertilizer was used at a rate of approximately 5.8–7.5 kg P ha^−1^ year^−1^. During maize growth, herbicides were not used, but pesticides were needed to control pest infestations. After the maize harvest, residues were left in the field without burning. While the field was left fallow until the next rainy season, cows were brought to the maize field to consume maize residues and grasses.

### 2.2. Experimental design and soil sampling

The CF-5Y (~180 m × 270 m) and M-5Y fields (~50 m × 78 m) were divided into three transects, starting from the uppermost slope to the lowermost slope. Along each transect in each field, five plots were designated at equal distances, and soil samples at a depth of 0–2 cm (surface layer) were collected. Previous studies (Higashida and Takao, 1985; Mehring et al., 2011; Liu C. et al., 2021; Barbour et al., 2022) have also reported that soil bacteria in the surface soil layer (0–2 cm) are more susceptible to changes in environmental conditions. Following this, the individual soil samples were combined to create a single composite sample for each transect. This approach was adopted due to the potential variation in soil nutrients caused by erosion processes along the slope (Arunrat et al., 2023b), which could impact the diversity of soil bacteria due to slope-related differences. Approximately 1 kg of soil was collected and placed in a plastic bag for analysis of the physical and chemical properties of the soil. The steel soil core (5.0 cm width × 5.5 cm length) was used to collect soil samples from each plot at a depth of 5 cm for bulk density analysis. Additionally, approximately 100 g of soil was collected, placed in zip-lock plastic bags, and kept at 10°C in the field and at −20°C in the laboratory for DNA extraction. Soil samplings were collected for a period of 1 year, at 3-month intervals, starting from March 2022 (summer season) and followed by June (rainy season), September (rainy season), December (winter season), and March 2023 (summer season). For each soil sampling plot during each time period, soil temperature and soil moisture were recorded at a depth of 2 cm using a Thermocouple Type K and a soil moisture meter, respectively. The results of soil temperature and soil moisture measurements are presented in Supplementary Table S2.

### 2.3. Soil physicochemical properties analysis

The soil bulk density was determined by weighing the dry soil sample in a steel soil core after it was dried at 105°C for 24 h. The soil texture was determined using the hydrometer method. Soil pH was measured using a pH meter with a 1:1 ratio of solid to water (National Soil Survey Center, 1996). Electrical conductivity (ECe) was measured by determining the saturation of paste extracts using an EC meter (USDA, 1954). The cation exchange capacity (CEC) was determined using the NH_4_OAc pH 7.0 method. Total nitrogen (TN) was analyzed using the micro-Kjeldahl method. Ammonium nitrogen (NH_4_-N) and nitrate-nitrogen (NO_3_-N) were measured using the KCl extraction method. The exchangeable calcium (exch.Ca), magnesium (exch.Mg), and potassium (exch.K) values were determined using atomic absorption spectrometry with NH_4_OAc pH 7.0 extraction. Available phosphorus (avail.P) was measured using the molybdate blue method (Bray II extraction) (Bray and Kurtz, 1945). Organic carbon (OC) was determined using potassium dichromate (K_2_Cr_2_O_7_) in sulfuric acid (Walkley and Black, 1934).

### 2.4. DNA extraction, bacterial 16 s amplification, and sequencing

Soil DNA was extracted using a DNeasy PowerSoil Pro DNA Kit (Qiagen). Subsequently, the DNA was amplified, targeting the V3–V4 region of the 16S rRNA gene, using primers 341F (5′-CCTAYGG-GDBGCWSCAG) and 805R (5′-GGACTAC-NVGGGTHTCTAAT-3′) (Klindworth et al., 2013). The amplicon was then subjected to a sequencing step using the paired-end Illumina Miseq platform at the Omics Sciences and Bioinformatics Center of Chulalongkorn University (Bangkok, Thailand).

### 2.5. Bioinformatics analysis

QIIME2 was used for bioinformatics analysis (Estaki et al., 2020). Forward and reverse primers were removed by Cutadapt (Martin, 2011). The sequences were then quality-filtered, merged, and chimera removed using the DADA2 plugin (Callahan et al., 2016). After this analysis, similar sequences were grouped together as amplicon sequence variants (ASVs). Then, the ASVs that contained less than two sequences were removed. Bacterial taxonomies were identified using the Silva v.138 database (Quast et al., 2013). Then, the ASVs that were identified as mitochondria or chloroplasts were removed. The remaining ASVs were then normalized to the smallest number of sequences from each sample using the rarefy plugin. The rarefied data were subjected to PICRUSt2 to predict enzyme abundance that was potentially performed by the bacteria found in this dataset (Douglas et al., 2020). Specifically, we highlight 13 enzymes involved in the soil system, such as those in the C, N, and P cycles (Das and Varma, 2011). These included acid phosphatase, alkaline phosphatase, alpha-N-acetylglucosaminidase, amidase, β-glucosidase, cellulase, chitinase, endo-1,4-β-xylanase, pectin lyase, rease, Xylan 1,4-β-xylosidase, nitrogenase, and nitrate reductase.

### 2.6. Statistical analysis

Statistical analyses were performed on PAST (Hammer et al., 2001) and R program (R Development Core, 2019). Beta diversity, which presents bacterial community composition, was visualized by Non-Metric Multidimensional Scaling (NMDS). The affected site and sampling times to bacterial community composition in both study sites were tested using two-way permutational multivariate analyses of variance (PERMANOVA). The correlation between soil properties and soil bacterial community compositions was analyzed using the Mantel test. Soil properties with significant correlation implied that these properties significantly shape the bacterial community composition in both study sites. The alpha diversity, presenting bacterial richness (observed richness and abundance-based coverage estimator (ACE) indexes) and bacterial diversity (Simpson and Shannon indexes), was computed using the microeco package (Liu X. et al., 2021). Differences between each alpha diversity index, relative abundance of top 10 genera, and abundance of soil enzymes were tested using ANOVA with repeated-measure. Spearman’s rank correlation was tested to determine the effect of soil properties on the abundance of bacterial genera and predictive enzymes.

## 3. Results

### 3.1. The effects of site and sampling time on bacterial community composition

**Figure 2:**
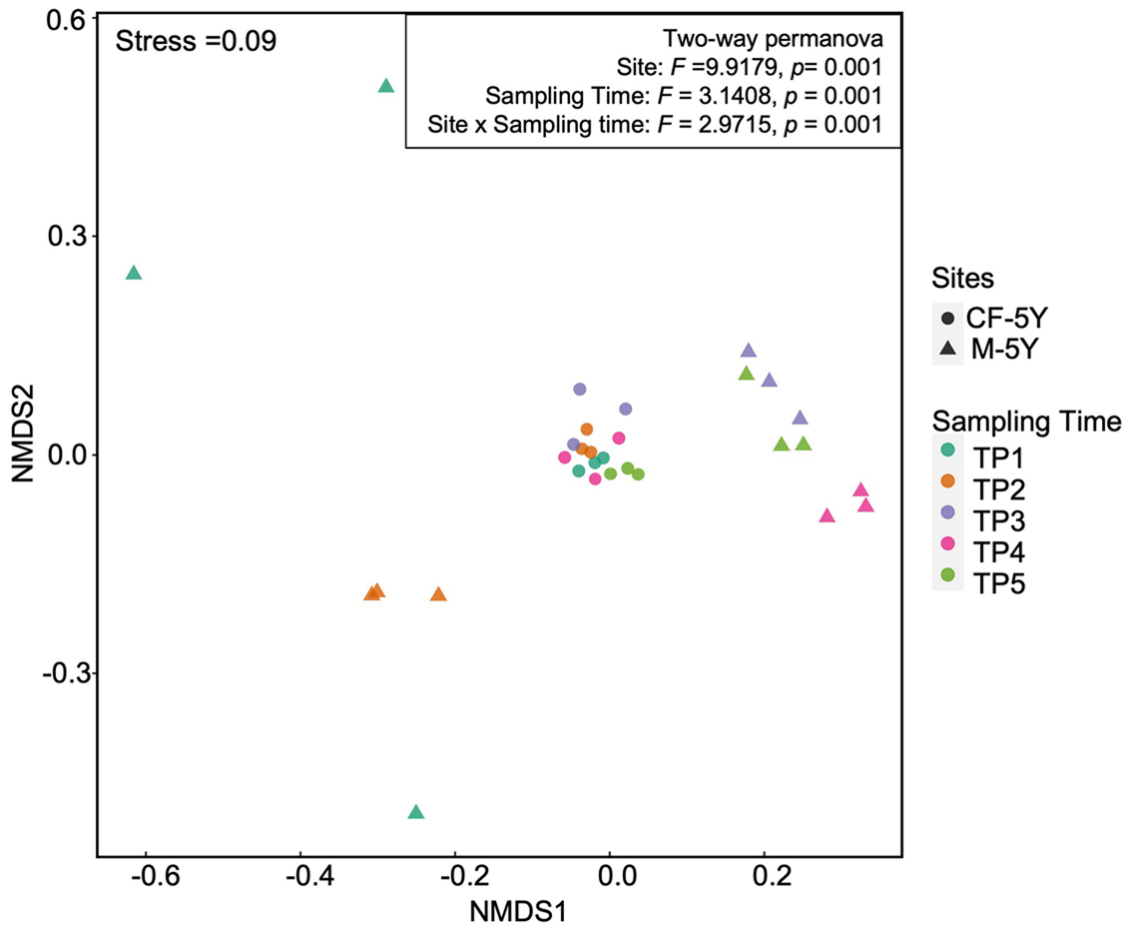
Bacterial community composition across all samples.

NMDS ordination of bacterial community composition in CF-5Y and M-5Y across all time points is presented in Figure 2. The bacterial community in CF-5Y was separated from M-5Y and the bacterial community at different sampling times. This indicated that the community composition of bacteria in CF-5Y was different from that in M-5Y. Two-way PERMANOVA analysis indicated that site, sampling time, and the interaction between these two factors significantly shape the bacterial community structure (*p* < 0.05; Figure 2). It indicated that CF-5Y and M-5Y fields, as well as the timing of sampling, were the vital factors, influencing the bacterial community structure. Soil physical and chemical properties of CF-5Y and M-5Y are presented in Tables 1, 2. There were relatively small variations in soil properties across seasons for both sites. This suggests that the soil properties remained relatively stable throughout the year. However, it is worth noting that OM and OC contents showed a declining trend over the seasons. TN, Avail. P, and Exch. K levels were highest during the rainy season (September), while Exch. Ca, Exch. Mg, NH_4_-N, and NO_3_-N peaked after the rainy season in December. The Mantel test revealed significant correlations between the bacterial community and soil properties, including OM, OC, Avail.P, Exch.Mg, TN, BD, sand, silt, and clay in M-5Y. The strongest correlations were observed in OM and OC, with correlation coefficients exceeding 0.5 (Table 3). However, no significant correlation was detected between soil properties and the bacterial community in CF-5Y (Table 3), likely due to the lower variation in soil properties across seasons.

**Table 1:** Variation in soil properties: bulk density (BD) (Mg m^−3^), electrical conductivity (EC_e_) (dS m^−1^), organic matter (OM) (%), organic carbon (OC) (%), total nitrogen (TN) (%), and proportion of sand, silt, and clay (%) with sites and time points.

Site	Season	Time point	pH (1:1)		EC_e_		BD		OM		OC		TN		%Sand		%Silt		%Clay	
			Mean	Std.	Mean	Std.	Mean	Std.	Mean	Std.	Mean	Std.	Mean	Std.	Mean	Std.	Mean	Std.	Mean	Std.
CF-5Y	Summer	March 2022	5.07	0.01	0.17	0.01	1.33^a^	0.01	8.80^a^	0.13	5.10^a^	0.08	0.33^a^	0.02	32.00	0.17	51.73^a^	1.45	16.28	1.28
	Rainy	June 2022	5.01	0.01	0.17	0.02	1.33^a^	0.01	8.65^a^	0.18	5.02^a^	0.10	0.35^a^	0.02	31.22	0.26	50.63	1.01	18.15	0.95
		September 2022	4.95	0.07	0.14	0.03	1.31^a^	0.01	7.69^a^	0.16	4.46^a^	0.09	0.37^a^	0.02	32.15	0.26	48.60^a^	0.97	19.25	0.91
	Winter	December 2022	4.76	0.11	0.15	0.01	1.32^a^	0.02	7.79^a^	0.06	4.52^a^	0.03	0.33^a^	0.02	33.75	0.28	45.19	0.90	21.06^a^	0.84
	Summer	March 2023	4.59	0.02	0.14	0.02	1.31^a^	0.01	7.55^a^	0.07	4.38^a^	0.04	0.34^a^	0.03	35.66	0.76	45.45	0.69	18.88^a^	0.57
M-5Y	Summer	March 2022	4.75	0.17	0.15	0.01	1.53^b^	0.01	2.35^b^	0.09	1.36^b^	0.05	0.17^b^	0.02	40.95	1.32	40.72^b^	1.45	18.33	0.34
	Rainy	June 2022	5.03	0.12	0.16	0.01	1.52^b^	0.02	2.35^b^	0.03	1.37^b^	0.01	0.19^b^	0.02	39.99	1.58	40.53	2.26	19.47	0.75
		September 2022	4.98	0.11	0.16	0.02	1.48^b^	0.02	2.14^b^	0.04	1.24^b^	0.02	0.21^b^	0.02	37.11	2.20	35.49^b^	3.61	27.40	1.99
	Winter	December 2022	4.61	0.04	0.17	0.02	1.45^b^	0.03	2.12^b^	0.02	1.23^b^	0.01	0.16^b^	0.01	26.98	1.76	41.88	2.11	31.13^b^	1.45
	Summer	March 2023	4.96	0.27	0.16	0.01	1.48^b^	0.01	2.08^b^	0.03	1.21^b^	0.02	0.18^b^	0.02	30.40	1.02	38.47	2.01	31.13^b^	1.45

^a,b^Significant statistical differences (*p* < 0.05) between CF-5Y and M-5Y fields.

**Table 2:** Variation in soil properties: cation exchange capacity (CEC) (meq 100 g^−1^), available P (mg kg^−1^), exchangeable K, Ca, and Mg (mg kg^−1^), NH_4_-N, and NO_3_-N (mg kg^−1^) contents with sites and time points.

Site	Season	Time point	CEC		Avail. P		Exch. K		Exch. Ca		Exch. Mg		NH_4_-N		NO_3_-N	
			Mean	Std.	Mean	Std.	Mean	Std.	Mean	Std.	Mean	Std.	Mean	Std.	Mean	Std.
CF-5Y	Summer	March 2022	18.48	0.45	6.19^a^	0.12	155.81	3.24	204.32^a^	0.56	97.90	1.29	17.77^a^	5.03	7.11^a^	0.00
	Rainy	June 2022	15.97	0.67	10.60^a^	0.31	177.15	4.19	214.73^a^	3.92	100.77^a^	2.04	14.21^a^	0.00	7.11^a^	0.00
		September 2022	14.80	0.64	15.12^a^	0.33	194.86	4.52	231.90^a^	4.23	108.82	2.20	14.21^a^	0.00	7.11^a^	0.00
	Winter	December 2022	14.97	0.15	5.82^a^	0.37	164.03	4.98	255.09	4.65	119.70	2.42	21.32	0.00	14.21	0.00
	Summer	March 2023	16.25	0.33	8.33^a^	0.39	172.30	8.38	255.09	4.65	117.24	5.71	21.32	0.00	14.21	0.00
M-5Y	Summer	March 2022	11.36	1.16	40.66^b^	1.89	171.79	6.56	306.66^b^	18.21	119.13	6.31	35.53^b^	0.00	28.42^b^	0.00
	Rainy	June 2022	14.40	0.00	41.89^b^	2.28	195.25	6.00	385.99^b^	27.98	203.33^b^	11.37	35.53^b^	0.00	35.53^b^	0.00
		September 2022	14.40	0.00	53.57^b^	1.70	232.39	19.76	348.57^b^	31.06	161.65	3.15	35.53^b^	0.00	35.53^b^	0.00
	Winter	December 2022	12.13	0.21	19.48^b^	0.76	183.87	3.67	289.73	24.46	148.46	1.90	28.42	0.00	14.21	0.00
	Summer	March 2023	11.20	0.57	37.82^b^	2.47	177.95	2.49	270.79	17.66	135.11	3.75	21.32	0.00	14.21	0.00

^a,b^Significant statistical differences (*p* < 0.05) between CF-5Y and M-5Y fields.

**Table 3:** The correlation coefficient and significant value of bacterial community and soil properties determined by the Mantel test.

Soil properties	CF-5Y		M-5Y	
	Correlation coefficient	Value of *p*	Correlation coefficient	Value of *p*
pH	0.1348	0.129	0.0739	0.205
OM	0.1212	0.136	0.6281	0.002*
OC	0.1308	0.098	0.6396	0.001*
ECe	0.1856	0.099	−0.0842	0.803
CEC	0.0255	0.417	0.1693	0.057
NH_4_-N	0.2252	0.008	0.0693	0.254
NO_3_-N	0.1883	0.045	0.1452	0.053
Avail.P	0.1282	0.122	0.2478	0.024*
Exch.K	0.1548	0.114	−0.0483	0.55
Exch.Ca	0.1670	0.045	0.0008	0.427
Exch.Mg	0.0681	0.209	0.4396	0.011*
Total.N	0.0185	0.390	0.3632	0.008*
BD	0.1551	0.117	0.4500	0.001*
Sand	0.1373	0.179	0.3514	0.004*
Silt	0.0668	0.247	−0.1987	0.934
Clay	−0.0551	0.609	0.5863	0.001*

### 3.2. Bacterial richness and diversity

**Figure 3:**
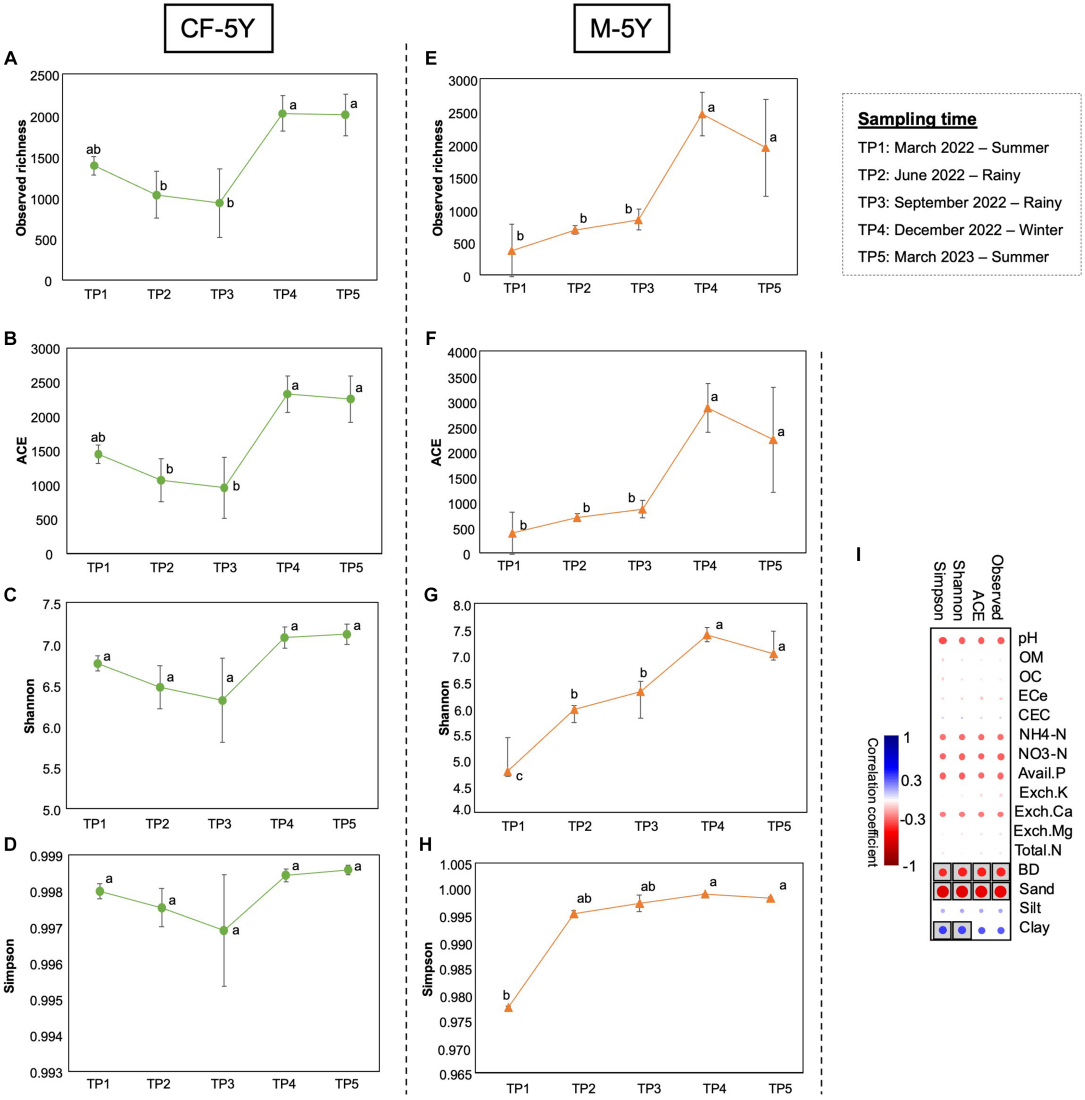
Alpha diversity indexes in CF-5Y **(A–D)** and M-5Y **(E-H)** across all time points. **(I)** Spearman’s rank correlation between the alpha diversity indexes and soil properties. Circle with box is the significant correlation (*p* < 0.05).

The alpha diversity index was used to assess the richness of bacteria, which included observed richness and ACE indexes. Moreover, the diversity of bacteria was evaluated using the Shannon and Simpson indexes. The richness and diversity indexes varied in different sampling times and study sites. In CF-5Y, all alpha diversity indexes dropped in TP2 and TP3 (rainy season) and increased during dry season (TP4—winter, TP5—summer). Significant differences were found in observed richness and ACE between TP3 and TP4, which indicate the transition from the rainy season to the winter season. The richness indexes significantly increased in this period (T4; Figures 3A–D). After 1 year of first sampling, all diversity indexes did not significantly change.

On the other hand, in M-5Y, all alpha diversity indexes were slightly increased in TP2 (rainy season); however, only a significant difference was found in the Shannon index. After that, observed richness, ACE, and Shannon indexes were significantly increased from TP3 to TP4, the transition between rainy and winter seasons. Then, these indexes did not change between TP4 and TP5, which are winter and summer seasons (Figures 3E–H). After 1 year of first sampling, all diversity indexes significantly increased.

Spearman’s rank correlation showed that the variation of alpha diversity indexes in both study sites was correlated with three soil properties, including BD, sand, and clay. Observed richness, ACE, Shannon, and Simpson indexes negatively correlated with BD and sand, whereas Shannon and Simpson indexes positively correlated with clay (Figure 3I).

### 3.3. Bacterial taxonomic distribution

A total of 37 phyla, 93 classes, 190 orders, 258 families, and 506 genera were detected in this study. The most abundant phyla across all samples were Actinobacteria, followed by Proteobacteria, Planctomycetes, and Chloroflexi.

#### 3.3.1. Bacterial taxonomic distribution in CF-5Y

**Figure 4:**
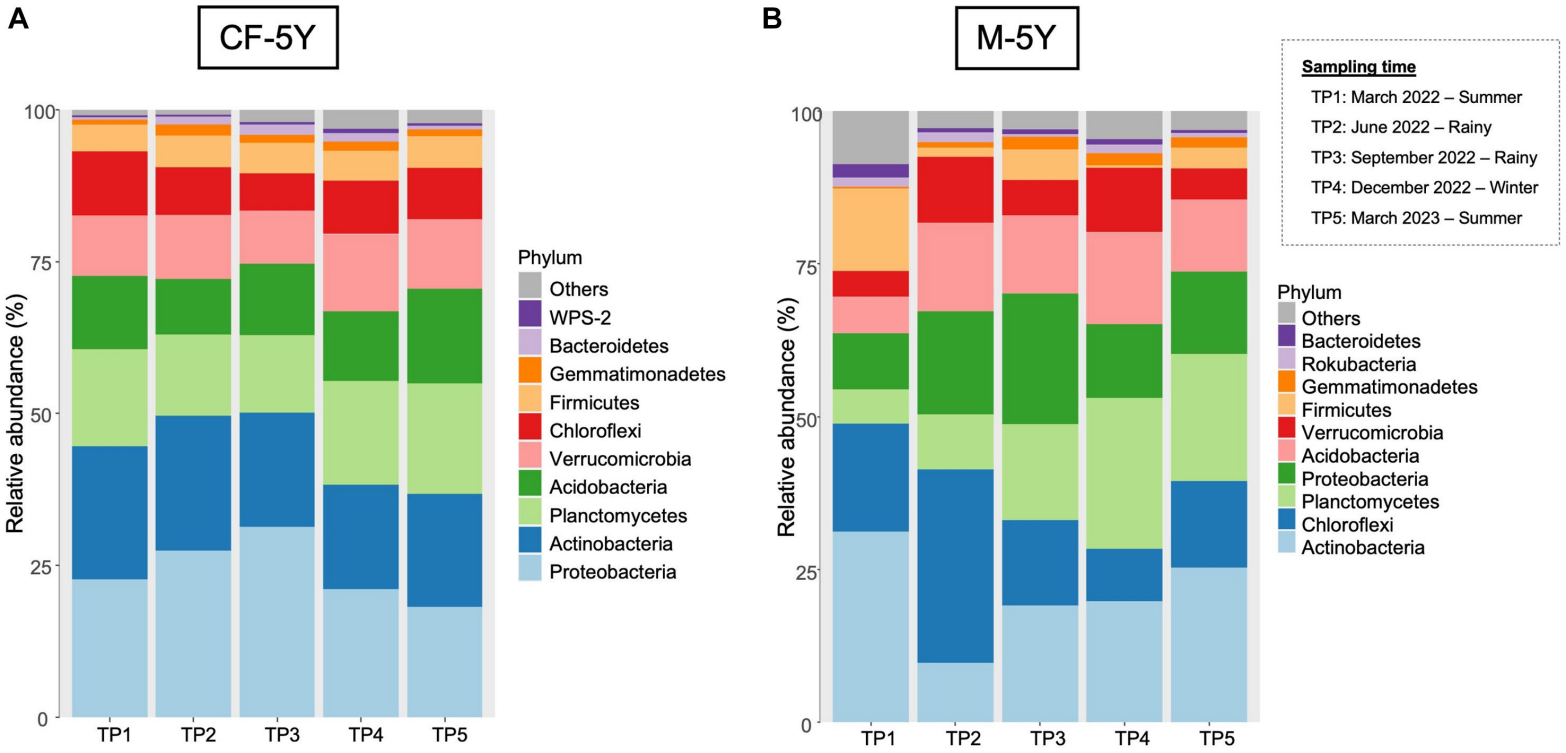
The most abundant bacterial phyla in **(A)** CF-5Y and **(B)** M-5Y.

The most abundant taxa in the first sampling time (TP1: March 2022—summer) were Proteobacteria (22.94%), followed by Actinobacteria (20.93%) and Planctomycetes (16.23%). Although there are some changes in the relative abundance in each taxon across all time points, the changes were small and did not affect the abundant group in each time point. For example, the abundance of proteobacteria increased to 25.91% (TP2) and 29.70% (TP3) in the rainy season and decreased to 24.90% (TP4) in winter and 18.46% (TP5) in summer. This phylum was still the most abundant phylum across all sampling points (Figure 4A).

**Figure 5:**
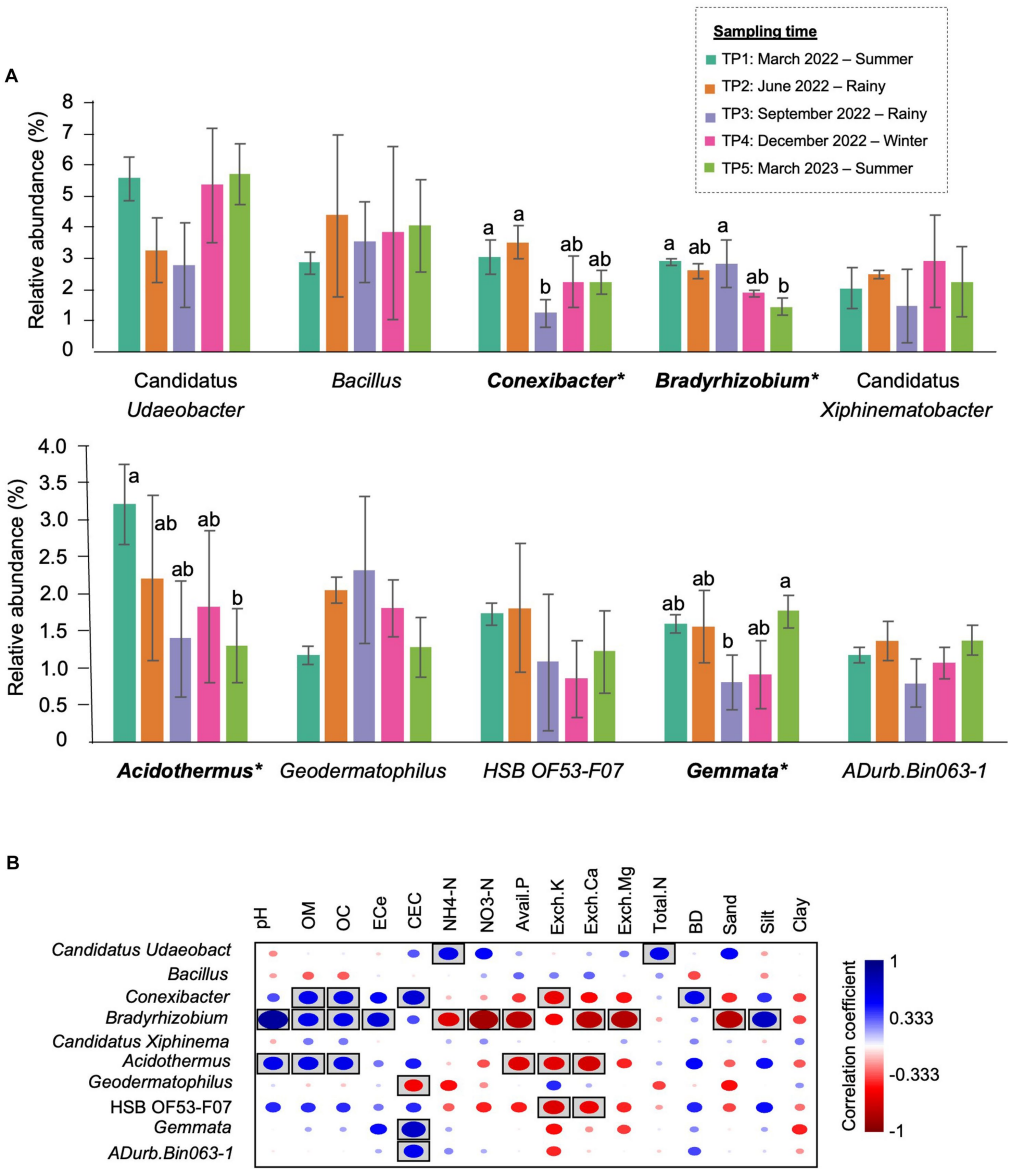
The most prevalent genera in CF-5Y and their correlation with soil properties. **(A)** The top 10 most abundant genera in CF-5Y. Taxa with asterisk (*) were statistically different between the sampling points (*p* < 0.05). **(B)** Spearman’s rank correlation between the abundant genera in CF-5Y and soil properties. Circle with box is the significant correlation (*p* < 0.05).

At the genus level, the most abundant genus in TP1 was Candidatus *Udaeobacter* (5.59%), followed by *Acidothermus* (3.21%) and *Conexibacter* (3.04%). In the rainy season, *Bacillus* became the most abundant genus, accounting for 4.39% of TP2 and 3.54% of TP3. Furthermore, the abundance of *Conexibacter* (from 3.04% in TP1 to 1.23% in TP3) and *Gemmata* (from 1.59% in TP1 to 0.80% in TP3) significantly decreased in this season. The most abundant genus in TP4 (winter) was Candidatus *Udaeobacter* (5.36%), followed by *Bacillus* (3.83%). In TP5 (summer- 1 year after the first sampling), the proportion of bacterial genera was changed compared with the first sampling point. Although Candidatus *Udaeobacter* (5.71%) was still the most abundant genus, the second most abundant genera were changed from *Acidothermus* to *Bacillus* (4.07%). Furthermore, the abundance of *Bradyrhizobium* significantly decreased after 1 year (Figure 5A).

Spearman’s rank correlation showed the correlation between soil properties and the relative abundance of bacterial genera. Here, we found that the significant genera, including *Bradyrhizobium, Acidothermus,* and *Conexibacter*, were positively correlated with OM and OC while negatively correlated with Avail.P, Exch.K, and Exch.Ca. On the other hand, *Gemmata* were positively correlated with CEC (Figure 5B).

#### 3.3.2. Bacterial taxonomic distribution in M-5Y

The relative abundance of bacterial phyla in M-5Y were notably varied at different time points. The most abundant taxa in the first sampling time (TP1: March 2022—summer) were Actinobacteria (31.18%) and Chloroflexi (17.71%). In TP2 (rainy season), the proportion of bacterial phyla notably changed. Chloroflexi became the most abundant phyla, accounting for 31.60%, followed by Proteobacteria (16.84%) and Acidobacteria (14.49%). After 3 months (TP3 rainy season), the most abundant phyla belonged to Proteobacteria (21.40%) and Actinobacteria (19.07%). In winter (TP4), the most abundant phyla belonged to Planctomycetes (24.71%) followed by Actinobacteria (19.19%). At the last sampling time which is 1 year after the first sampling, the relative abundance of bacterial phyla changed from the previous year. The most abundant phyla in TP5 were Actinobacteria (25.32%), followed by Planctomycetes (20.70%) and Chloroflexi (14.18%) (Figure 4B).

**Figure 6:**
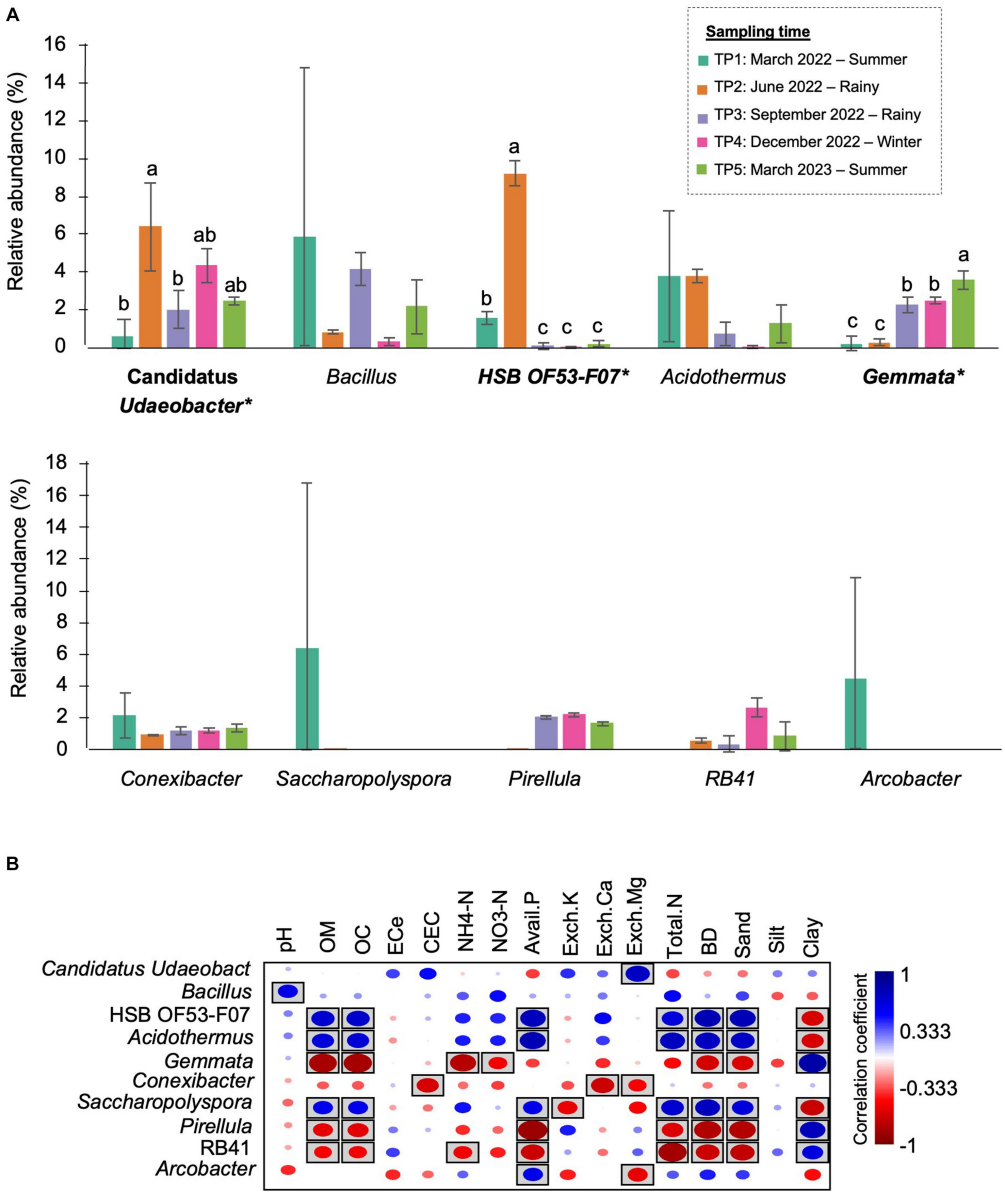
The most prevalent genera in M-5Y and their correlation with soil properties. **(A)** The top 10 most abundant genera in M-5Y. Taxa with asterisk (*) were statistically different between the sampling points (p < 0.05). **(B)** Spearman’s rank correlation between the abundant genera in M-5Y and soil properties. Circle with box is the significant correlation (*p* < 0.05).

At the genus level, *Bacillus* (5.89%), *Acidothermus* (3.84%), and *Conexibacter* (2.16%) dominated the bacterial community in TP1. *Arcobacter* were found only at this time point. In TP2 (rainy season), the abundance of HSB OF53-F07 (9.25%) and Candidatus *Udaeobacter* (6.43%) increased by approximately 8 and 6%, becoming the most abundant genera at this time point. After that, these two genera significantly decreased after 3 months (TP3). The most abundant genera in TP3 were *Bacillus* (5.89%) and *Gemmata* (2.29%). The latter significantly increased compared with TP1 and TP2. The most abundant genera in TP4 (winter) were Candidatus *Udaeobacter* (4.37%) and *Gemmata* (2.50%). In the last sampling time, *Gemmata* (3.61%) became the most abundant genus, followed by Candidatus *Udaeobacter* (2.47%) and *Bacillus* (2.19%) (Figure 6A). It is observed that the proportion of bacterial genera and the most abundant genera changed after 1 year.

Spearman’s rank correlation showed that the abundance of several genera were significantly correlated with soil properties. For example, Candidatus *Udaeobacter* was positively correlated with Exch. Mg. HSB OF53-F07, and *Acidodermus* was positively correlated with OM, OC, BD, and sand, while *Gemmata* was negatively correlated with those properties (Figure 6B).

### 3.4. Predictive function

**Figure 7:**
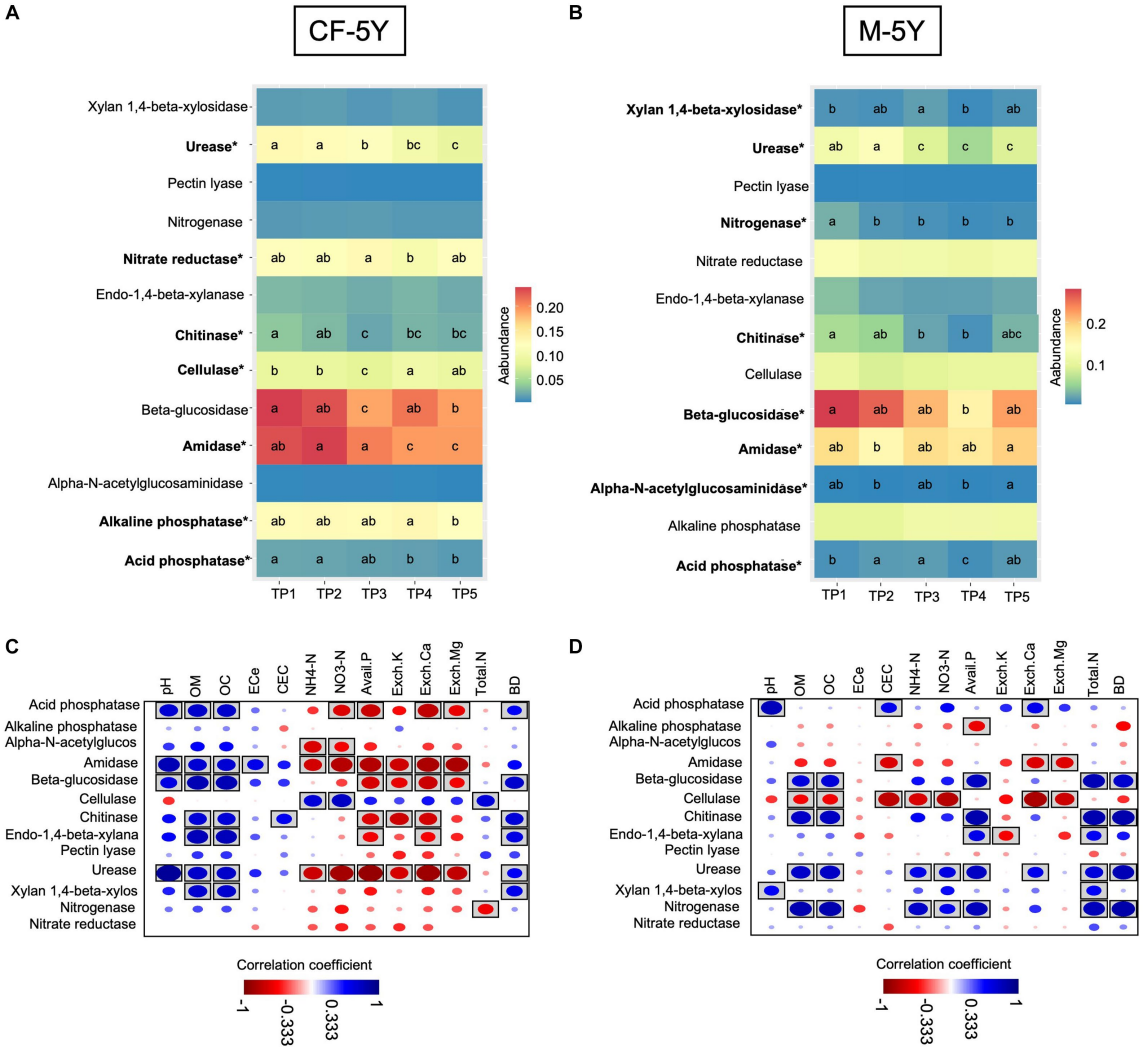
The abundance of soil enzyme predicted by PiCRUSt2 in **(A)** CF-5Y and **(B)** M-5Y and Spearman’s rank correlation between soil enzymes and soil properties in **(C)** CF-5Y and **(D)** M-5Y. Enzymes’ names with an asterisk (*) were statistically different between the sampling points (*p* < 0.05). Circle with box is the significant correlation (*p* < 0.05).

In this study, we used PICRUSt2 as a tool to predict bacterial-associated function and showed enzyme activity. Here, we found that in CF-5Y, urease, chitinase, cellulase, and β-glucosidase significantly decreased in TP3 (rainy season). After that, urease continuously decreased until TP5 (summer—1 year after the first sampling), becoming the least abundant across all time points. On the other hand, chitinase, cellulase, and β-glucosidase significantly increased in TP4 (winter) and TP5 (summer). The abundance of amidase did not change during the rainy season (TP3–TP4), but it significantly decreased in TP4 (winter) and TP5 (summer—1 year after the first sampling). The abundance of acid- and alkaline-phosphatase is quite stable, but it significantly decreases after 1 year of the first sampling (Figure 7A). Spearman’s rank correlation showed that chitinase, β-glucosidase, and urease positively correlated with OM and OC, while they negatively correlated with Avail. P, Exch. K, Exch. *Ca.* Cellulase positively correlated with NH_4_-N and NO_3_-N, whereas urease negatively correlated with these properties (Figure 7C).

In M-5Y, the abundance of chitinase, urease, and nitrogenase significantly decreased in the rainy season (TP2 or TP3). Chitinase slightly increased in TP5 (summer—1 year after the first sampling), but the urease and nitrogenase did not change. Their abundance is still low in TP4–TP5. On the other hand, xylan 1,4-β-xylosidase, and acid phosphatase significantly increased in the rainy season (TP2 and TP3), decreased in winter (TP4), then bounced back to a similar number of TP3 in summer (TP5). Overall, the abundance of these two enzymes in TP5 did not change from TP1 (Figure 7B). Spearman’s rank correlation showed that chitinase, usease, and nitrogenase positively correlated with OM, OC, NH_4_-N, NO_3_-N, total N, and BD, whereas xylan 1,4-β-xylosidase, and acid phosphatase positively correlated with pH (Figure 7D).

## 4. Discussion

### 4.1. Stability of soil bacteria in undisturbed soil and continuous maize cultivation

The soil bacterial community plays several important roles in soil functioning. Our study found that there was more variation in the richness and diversity of bacteria in M-5Y, whereas more stability was observed in CF-5Y (Figure 3).

The undisturbed soil over a 5-year period demonstrated more stability across seasons compared with M-5Y, as evidenced by the consistent values of diversity indexes between the TP1 and TP5 sampling times (Figure 3). Proteobacteria displayed a higher abundance in CF-5Y (Figure 4A), consistent with the findings of Wan and He (2020). Their study revealed that Proteobacteria were more prevalent in the soil of a natural forest in Northwest China, which was attributed to the nutrient-rich soil resulting from the rapid turnover of OM. Proteobacteria are generally classified as copiotrophic organisms, thriving and reproducing in nutrient-rich environments (Li et al., 2020; Zheng et al., 2020). It is evident that the availability of nutrients in CF-5Y primarily originated from the decomposition of OM, ranging from 7.55 to 8.80% across the seasons (Table 1).

Agricultural land management practices directly and indirectly affect soil environments, resulting in the alteration of activity and diversity of soil bacterial communities (Bending et al., 2002; Bulluck and Ristaino, 2002; Steenwerth et al., 2003). In cultivated land, higher soil microbial diversity can enhance soil fertility and maintain soil nutrient balance, thereby influencing crop growth (Turner et al., 2013). Zhang et al. (2021) reported that application fertilization, particularly nitrogen fertilizer, has a positive effect on maintaining the soil nutrients and increasing maize yield. Furthermore, tillage can enhance soil aeration and oxygen diffusion rates, potentially resulting in increased degradation of OM (Khan, 1996). This, in turn, could lead to elevated bacterial diversity and richness, along with enhanced soil enzyme activities (Zhou et al., 2019). Our study demonstrated that tillage practices in M-5Y during May had a notable impact, leading to a significant increase in the Shannon index from TP1 to TP2 (Figure 3G). This suggests that tillage practices could enhance both the number of bacteria species and the evenness of individual distribution among species, leading to increased diversity. The application of fertilizer in August significantly influenced the increase in both bacteria richness and diversity, as evidenced by a significant rise in observed richness and ACE indexes (Figures 3E,F), as well as the Shannon index from TP3 to TP4 (Figure 3G).

At 1 year of the first sampling (TP5), the richness and diversity significantly increased and higher than TP1 (Figure 3), assuming that the remaining synthetic nutrients support the increase in the abundance of soil bacteria. This is in line with the finding in Figure 4B that Actinobacteria were the most abundant in M-5Y. Similar to Proteobacteria, Actinobacteria are classified as copiotrophic with faster growth rates and rapidly increased under nitrogen-rich conditions in cropland soils (Fierer et al., 2012; Dai et al., 2018). Calderoli et al. (2017) reported that Actinobacteria are involved in nitrogen fixation through nitrogenase (*nifH*) gene expression.

Soil texture plays a crucial role in shaping soil bacterial taxonomic diversity and composition (Hemkemeyer et al., 2018). As shown in Figure 3I, clay content displayed a positive correlation with diversity indexes, while sand content and soil bulk density exhibited negative correlations with richness indexes. This observation aligns with the findings of Seaton et al. (2020) who indicated that clay and silt contents have a greater influence on soil bacterial composition compared with sand particles. Notably, clay content can provide microhabitats that facilitate organic matter stabilization, water retention, and the proliferation of soil bacteria biomass (Sparling, 1992; Aponte et al., 2014). In contrast, higher soil bulk density reduces soil porosity and limits water availability and aeration, which, in turn, may lead to a decrease in the abundance of soil bacteria (Jensen et al., 1996; Li et al., 2002).

Soil enzymes play a vital role in the decomposition of OM and the cycling of nutrients within ecosystems (Uwituze et al., 2022). β-Glucosidase was the most dominant enzyme in both CF-5Y and M-5Y sites (Figure 7). According to Wick et al. (2002), β-glucosidase is involved in the mineralization and cycling of carbohydrates in the soil. It catalyzes the hydrolysis of glycosidic bonds in cellobiose, resulting in the production of glucose (Chen et al., 2021). Eivazi and Tabatabai (1990) found positive correlations of β-glucosidase with OC concentration, which is in line with our study (Figures 7C,D). Amidase was considered the second dominant soil enzyme in both CF-5Y and M-5Y sites (Figure 7). Amidase enzyme is important in the nitrogen cycle, as it catalyzes the hydrolysis of amides to carboxylates and ammonia (Frankenberger and Tabatabai, 1980; Fournand and Arnaud, 2001). Our study showed lower amidase enzymes in M-5Y compared with CF-5Y sites (Figures 7A,B). We hypothesized that the utilization of synthetic fertilizers and pesticides in M-5Y could potentially inhibit certain soil bacterial communities, including those that play a role in amidase production. Moreover, M-5Y contained lower OM accumulation.

According to Frankenberger and Dick (1983), alkaline phosphatase, amidase, and catalase have been identified as good indicators of soil microbial activity and biomass. Our study is consistent with this finding, as both alkaline phosphatase and amidase enzymes dominated in both CF-5Y and M-5Y fields (Figures 7A,B). This suggests that the differences between the 5-year maize monoculture and 5-year undisturbed soil had no negative impact on soil bacterial communities and soil enzyme activity.

### 4.2. Seasonal influence on soil bacterial richness, diversity, and genera

Seasonal fluctuations can impact microbial diversity and richness (Samaritani et al., 2017; Keet et al., 2019) as changes in precipitation and temperature influence microbial activity and nutrient availability (Wu et al., 2020).

In the CF-5Y site, there was no significant change in bacterial diversity throughout the seasons (Figures 3C,D). However, a significant increase in bacterial richness was observed from the rainy season (TP3) to the winter season (TP4) (Figures 3A,B). On the other hand, a notable increase in the Shannon diversity index was observed from the summer season to the rainy season in M-5Y (Figure 3G). This increase could be attributed to the rise in soil moisture, which likely stimulated the growth of various bacterial genera. Moreover, the application of chemical fertilizer in August, coupled with the subsequent increase in soil moisture, could have facilitated the enhancement of bacterial richness (Figures 3E,F) and Shannon diversity indexes (Figure 3G). The increase in soil moisture is linked to an accelerated decomposition rate and the subsequent release of nutrients, thereby enhancing nutrient availability (Brockett et al., 2012; Madegwa and Uchida, 2021).

Tillage practices and reduced soil covering expose the soil surface to variations in weather conditions, leading to fluctuations in soil bacteria throughout seasonal changes. This study focused on a mountainous area, where the relationship between bacterial community structure and temperature fluctuations is significant. However, it is important to note that the effects of temperature on different taxonomic groups vary. For example, Firmicutes had a relatively high abundance in the summer but decreased in the winter season, while Acidobacteria showed the opposite trend (Figure 4B). The lower abundance of Firmicutes in winter may be attributed to reduced activity. The most prevalent genera in CF-5Y remained more stable throughout the seasons compared with M-5Y (Figures 5A, 6A). Meanwhile, some genera in M-5Y disappeared in certain seasons. Particularly, *Saccharopolyspora* and *Arcobacter* appeared only during the summer season (Figure 6A).

At the genus level, Candidatus *Udaeobacter* was identified as the most dominant during the summer season (TP1 and TP5), as well as in the winter season (TP4) in both CF-5Y and M-5Y sites. However, during the rainy season (TP2 and TP3), the dominant genus shifted to *Bacillus* in both CF-5Y and M-5Y sites (Figure 5A). Candidatus *Udaeobacter* belongs to the Verrucomicrobia phylum, which is composed of aerobic heterotrophs (Kadnikov et al., 2021). Candidatus *Udaeobacter* plays a role as amino acid and vitamin transporters, which involves sacrificing metabolic versatility in favor of storing surplus carbon as glycogen or starch. This alternative strategy contributes to its dominance in the soil environment even under resource-limiting conditions and high competition for labile carbon (Brewer et al., 2016). Huang et al. (2012) observed a decrease in the abundance of Verrucomicrobia, following an increase in available N, P, and K resulting from cotton straw application. Similarly, Lima et al. (2014) and Navarrete et al. (2015) found that higher verrucomicrobial community abundance is related to soils containing lower P and K contents. This may explain the abundance of Candidatus *Udaeobacter* during the summer and winter seasons, as these periods coincide with low available P and exchangeable K compared with the rainy season (Table 2). On the other hand, *Bacillus* sp. are the most dominant plant growth-promoting rhizobacteria (Tsotetsi et al., 2022), which colonize the area around plant roots and play a role in nutrient uptake (nitrogen fixation, solubilization, and mineralization of phosphorus and other nutrients) (Goswami et al., 2016). These abilities indicate that *Bacillus* can rapidly grow in nutrient-rich conditions during the rainy season. This is consistent with the observation of higher TN, available P, and exchangeable K during the rainy season compared with the summer and winter seasons (Tables 1, 2).

Seasonal variation in soil enzymes is driven by changes in soil moisture and temperature dynamics (Wallenstein et al., 2009; Machmuller et al., 2016). In our study, we observed a significant decrease in various soil enzymes during the rainy season in both CF-5Y (urease, chitinase, cellulase, and β-glucosidase) (Figure 7A) and M-5Y (chitinase, urease, and nitrogenase) sites (Figure 7B). This could be attributed to the effects of leaching and dilution. Heavy rainfall has the potential to leach soluble organic compounds and nutrients from the soil, causing these enzymes to be washed away from the active decomposition zone. Additionally, heavy rainfall can dilute the concentration of OM and nutrients in the soil, resulting in a reduced supply of substrates for the enzymes to act upon. Ladwig et al. (2015) reported that the rainy season could lead to a decrease in photosynthetic availability, potentially resulting in a reduced rate of expression of new enzymes compared with the loss of enzymes through decomposition processes. However, certain enzymes (chitinase, cellulase, and β-glucosidase) exhibited an increase during the summer seasons (Figures 7A,B), potentially attributed to the favorable combination of optimum temperature and soil moisture conditions. This is consistent with the study by Tomar and Baishya (2020), who reported that soil enzymes, with the exception of β-glucosidase activity, exhibited higher levels during the monsoon season in a semi-arid forest of Delhi, India, due to the optimum moisture and temperature conditions.

It is important to note that, unlike in a laboratory experiment, shifts in soil bacterial diversity can be driven by a combination of various physical factors, including land management practices, soil physicochemical properties, weather conditions, and vegetation cover. The presence and recovery of soil bacteria might have played important roles in the soil decomposition process, warranting further studies. The CF-5Y field, with its high soil moisture content (Table 1), could mitigate the fluctuations in daily and seasonal soil temperatures and potentially create a localized microclimate for soil bacteria. This, in turn, may contribute to the stability of the soil bacterial community throughout the changing seasons.

Our findings provide insights into the relative stability of soil bacteria in undisturbed soil compared with a maize cultivation field. Considering the context of climate change, with expectations of rising temperatures and shifting seasons in Northern Thailand, it is noteworthy that the dominant taxa in the bacterial communities of undisturbed soil and maize fields were found to be relatively similar. Therefore, it may be suggested that changing climate conditions may not lead to a significant shift in the soil bacterial community.

## 5. Conclusion

Our study found that undisturbed soil over a 5-year period exhibited more stability in the richness and diversity of bacteria across seasons compared with M-5Y. Fertilizer application and tillage practices in M-5Y influenced the diversity and richness of soil bacteria. Proteobacteria were the most abundant in CF-5Y, while Actinobacteria dominated in M-5Y. At the genus level, Candidatus *Udaeobacter* was the most dominant during the summer and winter seasons in both CF-5Y and M-5Y sites. However, during the rainy season, the dominant genus shifted to *Bacillus* in both CF-5Y and M-5Y fields. The soil bacterial community in M-5Y was strongly influenced by OM and OC, whereas in CF-5Y, there was no correlation between soil properties and the soil bacterial community. β-Glucosidase was the dominant enzyme in both CF-5Y and M-5Y sites, and it showed a positive correlation with OM and OC. Tillage practices and reduced soil covering in the M-5Y field exposed the soil surface to variations in weather conditions, leading to variations in soil bacteria throughout seasonal changes such as *Saccharopolyspora* and *Arcobacter,* which appeared only during the summer season. This study highlights that the shifts in soil bacterial diversity can be driven by a combination of land management practices, soil physicochemical properties, weather conditions, and vegetation cover. Longer time periods should be continuously investigated, as our study only covered a 1-year pattern of soil bacterial dynamics in these two environments.

## Data availability statement

The original contributions presented in the study are publicly available. This data can be found here: National Center for Biotechnology Information (NCBI) under the BioProject accession number: PRJNA1003674, https://www.ncbi.nlm.nih.gov/bioproject/PRJNA1003674.

## Author contributions

NA: Conceptualization, Data curation, Funding acquisition, Investigation, Methodology, Writing – original draft, Writing – review & editing. CS: Conceptualization, Methodology, Writing – original draft, Writing – review & editing. SS: Conceptualization, Investigation, Writing – original draft. RH: Conceptualization, Methodology, Supervision, Writing – original draft.

## References

[ref1] Aponte C. Matías L. González-Rodríguez V. Castro J. García L. V. Villar R.. (2014). Soil nutrients and microbial biomass in three contrasting Mediterranean forests. Plant Soil 2014, 57–72. doi: 10.1007/s11104-014-2061-5

[ref2] Arunrat N. Sereenonchai S. Hatano R. (2022b). Effects of fire on soil organic carbon, soil total nitrogen, and soil properties under rotational shifting cultivation in Northern Thailand. J. Environ. Manag. 302:113978. doi: 10.1016/j.jenvman.2021.113978 34710759

[ref3] Arunrat N. Sereenonchai S. Kongsurakan P. Hatano R. (2022a). Soil organic carbon and soil erodibility response to various land-use changes in Northern Thailand. Catena 219:106595. doi: 10.1016/j.catena.2022.106595

[ref4] Arunrat N. Sereenonchai S. Kongsurakan P. Iwai C. B. Yuttitham M. Hatano R. (2023a). Post-fire recovery of soil organic carbon, soil total nitrogen, soil nutrients, and soil erodibility in rotational shifting cultivation in Northern Thailand. Front. Environ. Sci. 11:1117427. doi: 10.3389/fenvs.2023.1117427

[ref5] Arunrat N. Sereenonchai S. Kongsurakan P. Yuttitham M. Hatano R. (2023b). Variations of soil properties and soil surface loss after fire in rotational shifting cultivation in Northern Thailand. Front. Environ. Sci. 11:1213181. doi: 10.3389/fenvs.2023.1213181

[ref6] Barbour K. M. Weihe C. Allison S. D. Martiny J. B. H. (2022). Bacterial community response to environmental change varies with depth in the surface soil. Soil Biol. Biochem. 172:108761. doi: 10.1016/j.soilbio.2022.108761, PMID: 15650683

[ref7] Barreiro A. Díaz-Raviña M. (2021). Fire impacts on soil microorganisms: mass, activity, and diversity. Curr. Opin. Environ. Sci. Health. 22:100264. doi: 10.1016/j.coesh.2021.100264

[ref8] Bending G. D. Turner M. K. Jones J. E. (2002). Interactions between crop residue and soil organic matter quality and the functional diversity of soil microbial communities. Soil Biol. Biochem. 34, 1073–1082. doi: 10.1016/S0038-0717(02)00040-8, PMID: 31316473

[ref9] Bray R. A. Kurtz L. T. (1945). Determination of total organic and available form of phosphorus in soil. Soil Sci. 59, 39–45. doi: 10.1097/00010694-194501000-00006, PMID: 37774876

[ref10] Brewer T. E. Handley K. M. Carini P. Gilbert J. A. Fierer N. (2016). Genome reduction in an abundant and ubiquitous soil bacterium *‘Candidatus* Udaeobacter copiosus’. Nat. Microbiol. 2:16198. doi: 10.1038/nmicrobiol.2016.198 27798560

[ref11] Brockett B. F. T. Prescott C. E. Grayston S. J. (2012). Soil moisture is the major factor influencing microbial community structure and enzyme activities across seven biogeoclimatic zones in western Canada. Soil Biol. Biochem. 44, 9–20. doi: 10.1016/j.soilbio.2011.09.003

[ref12] Bulluck L. R. Ristaino J. B. (2002). Effect of synthetic and organic soil fertility amendments on southern blight soil microbial communities and yield of processing tomatoes. Phytopathology 92, 181–189. doi: 10.1094/PHYTO.2002.92.2.181, PMID: 18943092

[ref13] Calderoli P. A. Collavino M. M. Behrends Kraemer F. Morrás H. J. M. Aguilar O. M. (2017). Analysis of *nif*H-RNA reveals phylotypes related to *Geobacteria* and cyanobacteria as important functional components of the N_2_-fixing community depending on depth and agricultural use of soil. MicrobiologyOpen 6:e502. doi: 10.1002/mbo3.502, PMID: 28766873 PMC5635172

[ref14] Callahan B. J. McMurdie P. J. Rosen M. J. Han A. W. Johnson A. J. A. Holmes S. P. (2016). DADA2: high-resolution sample inference from Illumina amplicon data. Nat. Methods 13, 581–583. doi: 10.1038/nmeth.3869, PMID: 27214047 PMC4927377

[ref15] Caon L. Vallejo V. R. Ritsema C. J. Geissen V. (2014). Effects of wildfire on soil nutrients in Mediterranean ecosystems. Earth Sci. Rev. 139, 47–58. doi: 10.1016/j.earscirev.2014.09.001, PMID: 37626979

[ref16] Certini G. (2005). Effects of fire on properties of forest soils: a review. Oecologia 143, 1–10. doi: 10.1007/s00442-004-1788-8, PMID: 15688212

[ref17] Chen A. Wang D. Ji R. Li J. Gu S. Tang R.. (2021). Structural and catalytic characterization of TsBGL, a β-glucosidase from Thermofilum sp. ex4484_79. Front. Microbiol. 12:723678. doi: 10.3389/fmicb.2021.781826, PMID: 34659150 PMC8517440

[ref18] Cordovez V. Dini-Andreote F. Carrión V. J. Raaijmakers J. M. (2019). Ecology and evolution of plant microbiomes. Annu. Rev. Microbiol. 73, 69–88. doi: 10.1146/annurev-micro-090817-062524, PMID: 31091418

[ref19] Dai Z. Su W. Chen H. Barberán A. Zhao H. Yu M.. (2018). Long-term nitrogen fertilization decreases bacterial diversity and favors the growth of actinobacteria and proteobacteria in agro-ecosystems across the globe. Glob. Chang. Biol. 24, 3452–3461. doi: 10.1111/gcb.14163, PMID: 29645398

[ref20] Das S. K. Varma A. (2011). “Role of enzymes in maintaining soil health” in Soil Enzymology. eds. Shukla G. Varma A., vol. 22 (Berlin/Heidelberg, Germany: Springer), 25–42.

[ref21] Douglas G. M. Maffei V. J. Zaneveld J. R. Yurgel S. N. Brown J. R. Taylor C. M.. (2020). PICRUSt2 for prediction of metagenome functions. Nat. Biotechnol. 38, 685–688. doi: 10.1038/s41587-020-0548-6, PMID: 32483366 PMC7365738

[ref22] Egamberdiyeva D. (2007). The effect of plant growth promoting bacteria on growth and nutrient uptake of maize in two different soils. Appl. Soil Ecol. 36, 184–189. doi: 10.1016/j.apsoil.2007.02.005, PMID: 37220675

[ref23] Eivazi F. Tabatabai M. A. (1990). Factors affecting glucosidase and galactosidase activities in soils. Soil Biol. Biochem. 22, 891–897. doi: 10.1016/0038-0717(90)90126-K, PMID: 16825450

[ref24] Ekasingh B. Gypmantasiri P. Thong-Ngam K. Grudloyma P. (2004). Maize in Thailand: Production Systems, Constraints, and Research Priorities. Mexico, DF: CIMMYT: International Maize and Wheat Improvement Center, Maize Production Systems Papers.

[ref25] Essel B. Abaidoo R. C. Opoku A. Ewusi-Mensah N. (2020). Economically optimal rate for nutrient application to maize in the semi-deciduous Forest zone of Ghana. J. Soil Sci. Plant Nutr. 20, 1703–1713. doi: 10.1007/s42729-020-00240-y, PMID: 33191974 PMC7655581

[ref26] Estaki M. Jiang L. Bokulich N. A. McDonald D. González A. Kosciolek T.. (2020). QIIME 2 enables comprehensive end-to-end analysis of diverse microbiome data and comparative studies with publicly available data. Curr. Protoc. Bioinformatics 70:e100. doi: 10.1002/cpbi.100, PMID: 32343490 PMC9285460

[ref27] Fierer N. Lauber C. L. Ramirez K. S. Zaneveld J. Bradford M. A. Knight R. (2012). Comparative metagenomic, phylogenetic and physiological analyses of soil microbial communities across nitrogen gradients. ISME J. 6, 1007–1017. doi: 10.1038/ismej.2011.159, PMID: 22134642 PMC3329107

[ref28] Fournand D. Arnaud A. (2001). Aliphatic and enantioselective amidases: from hydrolysisto acyl transfer activity. J. Appl. Microbiol. 91, 381–393. doi: 10.1046/j.1365-2672.2001.01378.x, PMID: 11556902

[ref29] Frankenberger W. T. Jr. Dick W. A. (1983). Relationships between enzyme activities and microbial growth and activity indices in soil. Soil Sci. Soc. Am. J. 47, 945–951. doi: 10.2136/sssaj1983.03615995004700050021x

[ref30] Frankenberger W. T. Jr. Tabatabai M. A. (1980). Amidase activity in soils: I. Method of assay. Soil Sci. Soc. Am. J. 44, 282–287. doi: 10.2136/sssaj1980.03615995004400020016x, PMID: 37456883

[ref31] Goswami D. Thakker J. N. Dhandhukia P. C. (2016). Portraying mechanics of plant growth promoting rhizobacteria (PGPR): a review. Cogent Food Agric 2:1127500. doi: 10.1080/23311932.2015.1127500

[ref32] Gouda S. Kerry R. G. Das G. Paramithiotis S. Shin H.-S. Patra J. K. (2018). Revitalization of plant growth promoting rhizobacteria for sustainable development in agriculture. Microbiol. Res. 206, 131–140. doi: 10.1016/j.micres.2017.08.016, PMID: 29146250

[ref33] Hammer O. Harper D. Ryan P. (2001). PAST: paleontological statistics software package for education and data analysis. Palaeontol. Electron. 4, 1–9.

[ref34] Hemkemeyer M. Dohrmann A. B. Christensen B. T. Tebbe C. C. (2018). Bacterial preferences for specific soil particle size fractions revealed by community analyses. Front. Microbiol. 9:149. doi: 10.3389/fmicb.2018.00149, PMID: 29527192 PMC5829042

[ref35] Higashida S. Takao K. (1985). Seasonal fluctuation patterns of microbial numbers in the surface soil of a grassland. Soil Sci. Plant Nutr. 31, 113–121. doi: 10.1080/17470765.1985.10555222

[ref36] Huang W. Bai Z. Hoefel D. Hu Q. Lv Q. Zhuang G.. (2012). Effects of cotton straw amendment on soil fertility and microbial communities. Front. Environ. Sci. Eng. 6, 336–349. doi: 10.1007/s11783-011-0337-z, PMID: 18943092

[ref37] Jensen L. S. McQueen D. J. Shepherd T. G. (1996). Effects of soil compaction on N mineralization and microbial-C and-NI field measurements. Soil Tillage Res. 38, 175–188. doi: 10.1016/S0167-1987(96)01033-1

[ref38] Kadnikov V. V. Mardanov A. V. Beletsky A. V. Grigoriev M. A. Karnachuk O. V. Ravin N. V. (2021). Thermophilic Chloroflexi dominate in the microbial community associated with coal-fire gas vents in the Kuznetsk Coal Basin, Russia. Microorganisms. 9:948. doi: 10.3390/microorganisms9050948, PMID: 33924824 PMC8146126

[ref39] Keet J. H. Ellis A. G. Hui C. Le Roux J. J. (2019). Strong spatial and temporal turnover of soil bacterial communities in South Africa’s hyperdiverse fynbos biome. Soil Biol. Biochem. 136:107541. doi: 10.1016/j.soilbio.2019.107541

[ref40] Khan A. R. (1996). Influence of tillage on soil aeration. J. Agron. Crop Sci. 177, 253–259. doi: 10.1111/j.1439-037X.1996.tb00243.x, PMID: 33016457

[ref41] Kim N. Zabaloy M. C. Guan K. Villamil M. B. (2020). Do cover crops benefit soil microbiome? A meta-analysis of current research. Soil Bio Biochem 142:107701. doi: 10.1016/j.soilbio.2019.107701

[ref42] Kleinman P. J. A. Pimentel D. Bryant R. B. (1995). The ecological sustainability of slash-and-burn agriculture. Agric. Ecosyst. Environ. 52, 235–249.

[ref43] Klindworth A. Pruesse E. Schweer T. Peplies J. Quast C. Horn M.. (2013). Evaluation of general 16S ribosomal RNA gene PCR primers for classical and next-generation sequencing-based diversity studies. Nucleic Acids Res. 41:e1. doi: 10.1093/nar/gks808, PMID: 22933715 PMC3592464

[ref44] Ladwig L. M. Sinsabaugh R. L. Collins S. L. Thomey M. L. (2015). Soil enzyme responses to varying rainfall regimes in Chihuahuan Desert soils. Ecosphere. 6:40. doi: 10.1890/ES14-00258.1

[ref45] Li S. F. Huang X. B. Lang X. D. Shen J. Su J. (2020). Cumulative effects of multiple biodiversity attributes and abiotic factors on ecosystem multifunctionality in the Jinsha river valley of southwestern China. For. Ecol. Manag. 472:118281. doi: 10.1016/j.foreco.2020.118281

[ref46] Li C. H. Ma B. L. Zhang T. Q. (2002). Soil bulk density effects on soil microbial populations and enzyme activities during the growth of maize (*Zea mays* L.) planted in large pots under field exposure. Can. J. Plant Sci. 82, 147–154. doi: 10.4141/S01-026

[ref47] Lima A. B. Cannavan F. S. Navarrete A. A. Teixeira W. G. Kuramae E. E. Tsai S. M. (2014). Amazonian dark earth and plant species from the Amazon region contribute to shape rhizosphere bacterial communities. Microb. Ecol. 69, 855–866. doi: 10.1007/s00248-014-0472-8 25103911

[ref48] Liu C. Cui Y. Li X. Yao M. (2021). Microeco: an R package for data mining in microbial community ecology. FEMS Microbiol. Ecol. 97:fiaa255. doi: 10.1093/femsec/fiaa255 33332530

[ref49] Liu X. Liu Y. Zhang L. Yin R. Wu G.-L. (2021). Bacterial contributions of bio-crusts and litter crusts to nutrient cycling in the mu us Sandy land. Catena 199:105090. doi: 10.1016/j.catena.2020.105090

[ref50] Machmuller M. B. Mohan J. E. Minucci J. M. Phillips C. A. Wurzburger N. (2016). Season, but not experimental warming, affects the activity and temperature sensitivity of extracellular enzymes. Biogeochemistry 131, 255–265. doi: 10.1007/s10533-016-0277-6, PMID: 37831294

[ref51] Madegwa Y. M. Uchida Y. (2021). Land use and season drive changes in soil microbial Communities and related functions in agricultural soils. Environmental DNA 3, 1214–1228. doi: 10.1002/edn3.244

[ref52] Martin M. (2011). Cutadapt removes adapter sequences from high-throughput sequencing reads. EMBnet.journal 17, 10–12. doi: 10.14806/ej.17.1.200, PMID: 28715235

[ref53] Mehring M. Glaser B. de Camargo P. B. Zech W. (2011). Impact of forest organic farming change on soil microbial C turnover using 13C of phospholipid fatty acids. Agronomy Sust. Developm. 31, 719–731. doi: 10.1007/s13593-011-0013-5

[ref54] Meng M. Lin J. Guo X. Liu X. Wu J. Zhao Y.. (2019). Impacts of forest conversion on soil bacterial community composition and diversity in subtropical forests. Catena 175, 167–173. doi: 10.1016/j.catena.2018.12.017

[ref55] Miah S. Dey S. Sirajul Haque S. M. (2010). Shifting cultivation effects on soil fungi and bacterial population in Chittagong Hill tracts, Bangladesh. J. For. Res. 21, 311–318. doi: 10.1007/s11676-010-0076-1

[ref56] Miah S. Haque S. M. S. Sumi W. Hossain M. M. (2014). Effects of shifting cultivation on biological and biochemical characteristics of soil microorganisms in Khagrachari hill district, Bangladesh. J. For. Res. 25, 689–694. doi: 10.1007/s11676-014-0453-2

[ref57] Moebius-Clune B. N. Van Es H. M. Idowu O. J. Schindelbeck R. R. Kimetu J. M. Ngoze S.. (2011). Long-term soil quality degradation along a cultivation chronosequence in western Kenya. Agric. Ecosyst. Environ. 141, 86–99. doi: 10.1016/j.agee.2011.02.018

[ref58] National Soil Survey Center. (1996). Soil Survey Laboratory Methods Manual. Soil Survey Investigations Report No. 42, Version 3.0, USDA. National Soil Survey Center Washington, DC, USA.

[ref59] Navarrete A. A. Diniz T. R. Braga L. P. P. Silva G. G. Z. Franchini J. C. Rossetto R.. (2015). Multi-analytical approach reveals potential microbial indicators in soil for sugarcane model systems. PLoS One 10:e0129765. doi: 10.1371/journal.pone.0129765, PMID: 26057123 PMC4461295

[ref60] Newbold T. Hudson L. N. Contu S. Hill S. L. L. Beck J. Liu Y.. (2018). Widespread winners and narrow-ranged losers: land use homogenizes biodiversity in local assemblages worldwide. PLoS Biol. 16:e2006841. doi: 10.1371/journal.pbio.2006841, PMID: 30513079 PMC6279023

[ref61] Pieterse C. M. J. de Jonge R. Berendsen R. L. (2016). The soil-borne supremacy. Trends Plant Sci. 21, 171–173. doi: 10.1016/j.tplants.2016.01.018, PMID: 26853594

[ref62] Quast C. Pruesse E. Yilmaz P. Gerken J. Schweer T. Yarza P.. (2013). The SILVA ribosomal RNA gene database project: improved data processing and web-based tools. Nucleic Acids Res. 41, D590–D596. doi: 10.1093/nar/gks1219, PMID: 23193283 PMC3531112

[ref63] R Development Core. (2019). A Language and Environment for Statistical Computing. R Foundation for Statistical Computing, Vienna, Austria, 1.

[ref64] Rerkasem K. Rerkasem B. (1995). Montane mainland South-East Asia: agroecosystems in transition. Glob. Environ. Change. 5, 313–322. doi: 10.1016/0959-3780(95)00065-V

[ref65] Samaritani E. Mitchell E. A. D. Rich J. Shrestha J. Fournier B. Frey B. (2017). Soil bacterial communities and ecosystem functioning change more strongly with season than habitat in a restored floodplain. Appl. Soil Ecol. 112, 71–78. doi: 10.1016/j.apsoil.2016.12.010

[ref66] Sarkar D. Meitei C. B. Baishya L. K. Das A. Ghosh S. Chongloi K. L.. (2015). Potential of fallow chronosequence in shifting cultivation to conserve soil organic carbon in Northeast India. Catena 135, 321–327. doi: 10.1016/j.catena.2015.08.012

[ref89] Schloter M. Dilly O. Munch J. C. (2003). Indicators for evaluating soil quality. Agric. Ecosyst. Environ. 98, 255–262. doi: 10.1016/S0167-8809(03)00085-9

[ref67] Schulz S. Brankatschk R. Dümig A. Kögel-Knabner I. Schloter M. Zeyer J. (2013). The role of microorganisms at different stages of ecosystem development for soil formation. Biogeosciences 10, 3983–3996. doi: 10.5194/bg-10-3983-2013, PMID: 36632968

[ref68] Seaton F. M. George P. B. Lebron I. Jones D. L. Creer S. Robinson D. A. (2020). Soil textural heterogeneity impacts bacterial but not fungal diversity. Soil Biol. Biochem. 144:107766. doi: 10.1016/j.soilbio.2020.107766, PMID: 32361439

[ref69] Shahzad T. Rashid M. I. Maire V. Barot S. Perveen N. Alvarez G.. (2018). Root penetration in deep soil layers stimulates mineralization of millennia-old organic carbon. Soil Biol. Biochem. 124, 150–160. doi: 10.1016/j.soilbio.2018.06.010

[ref70] Smith N. R. Kishchuk B. E. Mohn W. W. (2008). Effects of wildfire and harvest disturbances on Forest soil bacterial communities. Appl. Environ. Microbiol. 74, 216–224. doi: 10.1128/AEM.01355-07, PMID: 18024684 PMC2223229

[ref71] Sparling G. (1992). Ratio of microbial biomass carbon to soil organic carbon as a sensitive indicator of changes in soil organic matter. Soil Res. 30, 195–207. doi: 10.1071/SR9920195, PMID: 36297788

[ref72] Steenwerth K. L. Jackson L. E. Calderón F. J. Stromberg M. R. Scow K. M. (2003). Soil microbial community composition and land use history in cultivated and grassland ecosystems of coastal California. Soil Biol. Biochem. 35, 489–500. doi: 10.1016/S0038-0717(03)00028-2

[ref73] Szoboszlay M. Dohrmann A. B. Poeplau C. Don A. Tebbe C. C. (2017). Impact of land-use change and soil organic carbon quality on microbial diversity in soils across Europe. FEMS Microbiol. Ecol. 93:fix146. doi: 10.1093/femsec/fix146, PMID: 29087486

[ref74] Tomar U. Baishya R. (2020). Seasonality and moisture regime control soil respiration, enzyme activities, and soil microbial biomass carbon in a semi-arid forest of Delhi, India. Ecol. Proces. 9:50. doi: 10.1186/s13717-020-00252-7

[ref75] Tsotetsi T. Nephali L. Malebe M. Tugizimana F. (2022). Bacillus for plant growth promotion and stress resilience: what have we learned? Plan. Theory 11:2482. doi: 10.3390/plants11192482, PMID: 36235347 PMC9571655

[ref76] Turner T. R. James E. K. Poole P. S. (2013). The plant microbiome. Genome Biol 14:209. doi: 10.1186/gb-2013-14-6-209 23805896 PMC3706808

[ref77] USDA. (1954). Diagnosis and Improvement of Saline and Alkali Soils, Agriculture. Handbook No. 60, U.S. Salinity Laboratory. Government Printing Office, Washington, DC.

[ref78] Uwituze Y. Nyiraneza J. Fraser T. D. Dessureaut-Rompré J. Ziadi N. Lafond J. (2022). Carbon, nitrogen, phosphorus, and extracellular soil enzyme responses to different land use. Front. Soil Sci. 2:814554. doi: 10.3389/fsoil.2022.814554, PMID: 34346741

[ref79] Walkley A. Black J. A. (1934). An examination of the dichormate method for determining soil organic matter and a proposed modification of the chromic acid titration method. Soil Sci. 37, 29–32. doi: 10.1097/00010694-193401000-00003

[ref80] Wallenstein M. D. McMahon S. K. Schimel J. P. (2009). Seasonal variation in enzyme activities and temperature sensitivities in Arctic tundra soils. Glob. Chang. Biol. 15, 1631–1639. doi: 10.1111/j.1365-2486.2008.01819.x

[ref81] Wan P. He R. (2020). Soil microbial community characteristics under different vegetation types at the national nature reserve of Xiaolongshan Mountains, Northwest China. Eco. Inform. 55:101020. doi: 10.1016/j.ecoinf.2019.101020

[ref82] Wapongnungsang O. E. Upadhyay K. K. Tripathi S. K. (2021). Soil fertility and rice productivity in shifting cultivation: impact of fallow lengths and soil amendments in Lengpui, Mizoram Northeast India. Heliyon. 7:e06834. doi: 10.1016/j.heliyon.2021.e06834, PMID: 33981893 PMC8082545

[ref83] Whitbread A. Blair G. Naklang K. Lefroy R. Wonprasaid S. Konboon Y.. (1999). The management of rice straw, fertilisers and leaf litters in rice cropping systems in Northeast Thailand: 2. Rice yields and nutrient balances. Plant Soil 209, 21–28.

[ref84] Wick B. Kühne R. F. Vielhauer K. Vlek P. L. (2002). Temporal variability of selected soil microbiological and biochemical indicators under different soil quality conditions in South-Western Nigeria. Biol Fert Soils. 35, 155–167. doi: 10.1007/s00374-002-0455-7

[ref85] Williams D. R. Alvarado F. Green R. E. Manica A. Phalan B. Balmford A. (2017). Land-use strategies to balance livestock production, biodiversity conservation and carbon storage in Yucatan, Mexico. Glob. Chang. Biol. 23, 5260–5272. doi: 10.1111/gcb.13791, PMID: 28614629

[ref86] Wu J. Sha C. Wang M. Ye C. Li P. Huang S. (2021). Effect of organic fertilizer on soil bacteria in maize fields. Land. 10:328. doi: 10.3390/land10030328, PMID: 37800353

[ref87] Wu K. Xu W. Yang W. (2020). Effects of precipitation changes on soil bacterial community composition and diversity in the Junggar desert of Xinjiang China. PeerJ. 8:e8433. doi: 10.7717/peerj.10403, PMID: 32025376 PMC6991129

[ref88] Yang Z. Xu Y. Li H. Li S. Wang X. Chai H. (2022). Difference of bacterial community structure in the meadow, maize, and continuous cropped alfalfa in Northeast China. Front. Microbiol. 13:794848. doi: 10.3389/fmicb.2022.1082025, PMID: 35432280 PMC9008367

[ref90] Zhang M. Zhang X. Zhang L. Zeng L. Liu Y. Wang X.. (2021). The stronger impact of inorganic nitrogen fertilization on soil bacterial community than organic fertilization in short-term condition. Geoderma 382:114752. doi: 10.1016/j.geoderma.2020.114752

[ref91] Zheng X. Lin C. Guo B. Yu J. Ding H. Peng S.. (2020). Effects of re-vegetation restoration on soil bacterial community structure in degraded land in subtropical China. Eur. J. Soil Biol. 98:103184. doi: 10.1016/j.ejsobi.2020.103184

[ref92] Zhou Y. Zhou B. Xu F. Muhammad T. Li Y. (2019). Appropriate dissolved oxygen concentration and application stage of micro-nano bubble water oxygation in greenhouse crop plantation Agric. Water Manag. 223:105713. doi: 10.1016/j.agwat.2019.105713

