# Supplementary material for: Stability of soil bacteria in undisturbed soil and continuous maize cultivation in Northern Thailand
Source: Front Microbiol. 2023 Nov 3;14:1285445. doi: 10.3389/fmicb.2023.1285445 (PMC10655093; doi:10.3389/fmicb.2023.1285445)
Supplement: Supplementary file 1 [file Data_Sheet_1.docx]

**Supplementary material**

**Table S1.** Average weather data during March 2022 to March 2023

| **Year** | **2022** | | | | | | | | | | | **2023** | | |
| --- | --- | --- | --- | --- | --- | --- | --- | --- | --- | --- | --- | --- | --- | --- |
| **Sampling time points** | **TP1** |  |  | **TP2** |  |  | **TP3** |  |  | **TP4** |  | |  | **TP5** |
| **Month** | **March** | **April** | **May** | **June** | **July** | **August** | **September** | **October** | **November** | **December** | **January** | | **February** | **March** |
| **Variables** |  | | | | | | | | | | | | | |
| Maximum temperature (°C) | 31.1 | 30.1 | 28.8 | 29.6 | 28.8 | 27.8 | 27.2 | 26.6 | 27.2 | 24.9 | 25.9 | | 27.5 | 32.3 |
| Minimum temperature (°C) | 22.3 | 22.1 | 21.8 | 22.5 | 23.1 | 22.7 | 22.1 | 21.4 | 20.5 | 18.6 | 16.8 | | 19.0 | 20.0 |
| Mean temperature (°C) | 21.1 | 21.1 | 21.5 | 21.6 | 22.3 | 22.0 | 21.4 | 20.1 | 19.6 | 17.6 | 16.4 | | 17.0 | 25.4 |
| Precipitation (mm) | 57.4 | 177.5 | 351.0 | 141.9 | 269.9 | 420.9 | 525.6 | 215.4 | 30.5 | 37.6 | 41.6 | | 25.0 | 6.8 |
| Number of rain day (days) | 7.0 | 11.5 | 19.5 | 14.5 | 23.0 | 25.5 | 22.0 | 12.0 | 6.0 | 1.5 | 4.0 | | 3.5 | 1.5 |

Sources: Thai Meteorological Department, sourced from Doi Ang Khang and Mueang Chiang Mai stations

**
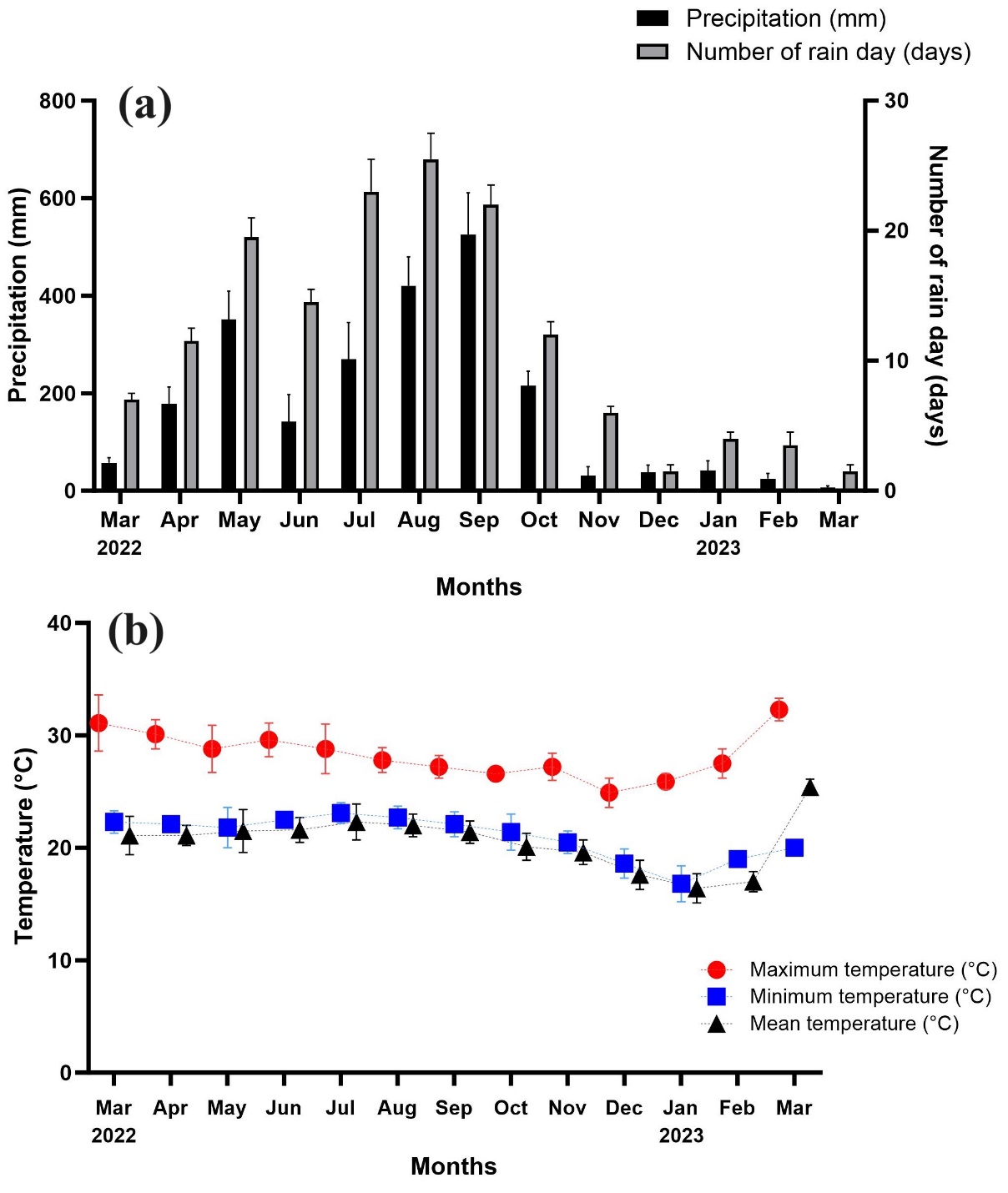
**

**Figure S1.** Variation of weather data during March 2022 to March 2023

**Table S2.** Soil moisture and soil temperature of study sites.

| **Sites** | **Variables** | **Time points** | | | | |
| --- | --- | --- | --- | --- | --- | --- |
|  |  | TP1 | TP2 | TP3 | TP4 | TP5 |
| CF-5Y | Soil moisture (%) | 35.1±5.3^b^ | 43.5±4.5^a^ | 44.5±5.5^a^ | 38.9±5.1^b^ | 36.2±5.0^b^ |
|  | Soil temperature (°C) | 24.5±2.0^a^ | 23.5±3.0^a^ | 22.3±3.0^a^ | 22.1±2.0^a^ | 25.1±2.0^a^ |
| M-5Y | Soil moisture (%) | 28.3±4.0^b^ | 38.5±5.0^a^ | 41.5±4.5^a^ | 35.5±6.5^b^ | 29.3±3.5^b^ |
|  | Soil temperature (°C) | 26.8±2.0^a^ | 25.5±3.0^a^ | 24.5±3.5^a^ | 23.5±3.0^a^ | 27.5±3.5^a^ |

a-b, significant statistical differences (*p* <0.05). TP1= March 2022 (summer), TP2= June 2022 (rainy), TP3= September 2022 (rainy), TP4= December 2022 (winter), TP5= March 2023 (summer)
